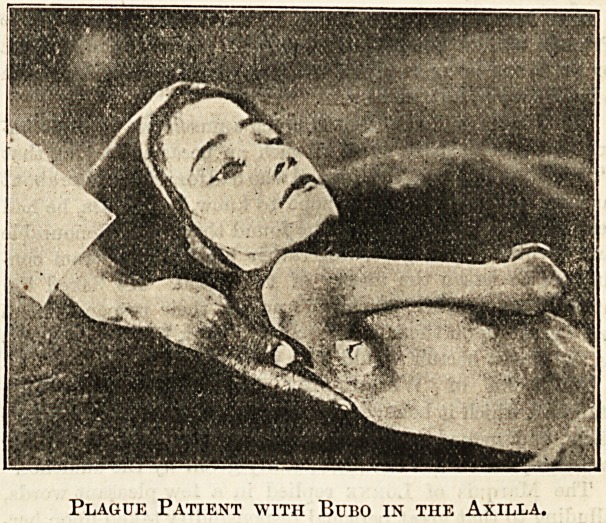# "The Hospital" Nursing Mirror

**Published:** 1899-07-08

**Authors:** 


"The Hospital" Nursing Mirror.

Being the Nursing Section of "The Hospital."

[Contributions for this Section of "The Hospital" should be addressed to the Editor, The Hospital, 28 & 29, Southampton Street, Strand, London, W.C., and should have the word "Nursing" plainly written in left-hand top corner of the envelope.]

### Notes on News from the Nursing World.

### THE VISITING COMMITTEE OF THE PRINCE OF WALES'S FUND AND HOSPITAL NURSES.

Sir Savile Crossley has, happily, completely disposed of the idea entertained by Mr. Sydney Holland that visitors to hospitals from the Prince of Wales's Fund ask no questions "either *viva voce* or on the forms, as to the training of nurses, or as to their hours of duty, their sleeping accommodation, or their food." Far from this being the case, Sir Savile points out that "on the forms used by all the visitors of the Fund special questions are asked as to the nurses employed and the accommodation for them," and he mentions that "the provision for nurses at the London Hospital last year formed one of the principal questions examined by the visitors and reported on by them." This is really also an answer to the appeal of Mr. J. G. Talbot, who, having stated "that it has been represented to him in several quarters that the food of the nurses in some of the hospitals is deficient both in quality and in quantity," begs the committee of the Prince's Fund to make careful investigations into this subject. Mr. Talbot, while justly affirming that the nursing of the sick poor has, in our own times, "enormously advanced and improved," adds, "it must not be said that the very agents whom we employ in that improvement are themselves the victims of neglect." We are confident that if in the course of their inquiries the visitors of the Fund find any evidence in any particular case that the nurses are the victims of neglect they will insist upon reform before a grant is made or renewed.

### PROPOSED PETITION TO PARLIAMENT AGAINST THE EMPLOYMENT OF TRAINED NURSES.

It was seriously proposed at a meeting of the Limavady Board of Guardians—perhaps we ought to say that Limavady is in Ireland—to petition Parliament to restrain the powers of the Local Government Board because the latter insisted upon the appointment by the Guardians of a trained nurse. The author of this proposition declared at a meeting of Guardians that if the matter were one between himself individually and the Local Government Board, "before he would submit to the indignity of such an order he would put on his hat and never come near the place again." It is almost a pity that the headgear was not at once resorted to, for, though the owner afterwards urged submission to the order, his suggestion to appeal to Parliament is ridiculous. The House of Commons may make blunders, but it is not likely to attempt to cripple the power of the Local Government Board when the latter is exercised in the interests of the community. Nor is there any case for the alternative proposition, that a court of appeal should be constituted to decide differences between boards of guardians and the Local Government Board. The Local Government Board, like all other State departments, can always be arraigned at the bar of public opinion if it does wrong; but most persons will hold that in "forcing trained nurses on boards of guardians," whether in Ulster or elsewhere, it is simply doing its duty under the Act of Parliament from which it derives authority.

### WISE WORDS FROM A BISHOP'S WIFE.

Thanks to the courtesy of the Bishop of Bangor a bazaar has been held at the Palace in the cathedral city on behalf of the Bangor Nursing Institute. Mrs. Williams, the Bishop's wife, in performing the opening ceremony, made some excellent remarks about the importance of putting the institute on a firm financial footing. "That," she said, "is far more desirable than even having such delightful gatherings as the one in which I am participating." And she expressed the hope that in future no sales of work would be wanted to keep the institute going. There is reason to believe that her appeal for "the more lasting benefit of regular subscriptions" did not fall on deaf ears.

### NURSING IN CYPRUS.

There are no English nurses at present at the hospital in Larnaca, but a new building is being erected, and it is hoped, when it is finished, that the services of one English trained nurse will be secured. At the other hospital in Micosia there is an English nurse as well as two native nurses, the staff of the institution also including a chief medical British officer, a non-resident surgeon, and two Police Yaptishs as attendants. The hospital is situated in a large compound about a quarter of a mile outside the Papho Gate, and consists of two blocks, one of which is used for medical officers' offices, British nurses' quarters, private patients' room, and operation-room. The other block originally had 22 beds, but in 1897 a wing was added, containing eight small wards of two beds each, meant for eye cases, which in Cyprus are numerous. The hospital was built in 1889, and has proved a great blessing to the poor. The sick who cannot ride on mules or donkeys are conveyed to it by bullock-waggon. The last two years the matron managed to have a Christmas tree for the children, which proved a treat to everyone.

### GUY'S HOSPITAL LADIES' ASSOCIATION.

The report of this excellent association shows it to be in a flourishing condition. The number of its members has increased by 70 in the last year, and four new branches are being formed. These country and suburban branches do excellent work in widening the circle of those interested in the hospital, and they, as well as the London branch, hold work parties to make garments for the hospital. They also send old clothes, flowers and fruit, &c., for the use of the patients. Any lover of Guy's may help the work by forming a branch in her own neighbourhood, or by joining the Central association. For the past year the association has sent £50 to the hospital treasurer in support of a bed in the maternity ward. It is satisfactory to hear that this year they will hand over £100 to support two beds in the same ward.

### AT THE NIGHTINGALE HOME.

One of the pleasantest annual hospital entertainments is the garden party given by the secretary of the Nightingale Fund and Mrs. Bonham Carter at St. Thomas's Hospital. On Saturday many visitors gathered in the Nightingale Home, and though unfortunately the day was too rough and stormy for the splendid stretch of river terrace to be used much as a promenade, the corridor made a good substitute. Miss Gordon and Mr. and Mrs. Bonham Carter received the guests in the dining-hall, where tea was laid. Ices were served in the corridor, which was prettily arranged with plants and seats; and the band played just outside. Amongst the guests were Miss Amy Hughes, Miss Gray, Miss Smedley, Miss H. A. C. Gordon, Miss Vincent, and several members of the medical staff. The wards were gay with flowers, and the newly-opened City of London Ward, with its very up-to-date appointments and fittings, came in for a due share of admiration.

### THE QUESTION OF A NURSES' HOME AT CHESTER.

In spite of a certain amount of opposition, the Chester Board of Guardians have decided to prepare a scheme for the erection of a nurses' home within the workhouse grounds. This will enable them to carry out the new system, under which the staff of nurses in the union hospital will consist of one superintendent, four trained nurses, and four probationers—the latter receiving nominal salaries. It is probable that the determination to erect the home was arrived at in consequence of the difficulty which has been experienced in obtaining nurses. Mr. Rowe Morris, one of the strongest advocates of the home, said "that vacancies for nurses were perpetual, and that the guardians could hardly get any response to their advertisements." We are glad to hear it, for the best way of compelling local authorities to provide proper accommodation for nurses is for the latter to refuse to take service under bodies which have the reputation of being offenders in this respect. When it is known that there is to be a home for the nursing staff under a trained superintendent, the Chester Guardians' advertisements will doubtless attract sufficient replies.

### THE HARD CASE OF NURSE CAVARET.

We hope the Local Government Board will allow generous treatment to be meted out to Nurse Cavaret. The proposal made by the committee of the North-Eastern Hospital does not, however, quite merit this description. Nurse Cavaret contracted scarlet fever six weeks after she entered the North-Eastern Hospital as second-class assistant nurse, and subsequently developed Bright's disease. She was never able to resume duty, and in March, 1898, the medical superintendent having reported that "she would probably never be fit for hospital work again," arrangements were made for her admission into Guy's Hospital. After a stay of nine months she has now been discharged. On her appointment at the North-Eastern she gave notice that she did not intend to avail herself of the provisions of the Poor Law Officers' Superannuation Act, and she is therefore not entitled to any gratuity under it. But the committee of the hospital went so far as to recommend a grant of £30. Several of the members of the Metropolitan Board of Works, before whom the case was brought on Saturday, urged that the amount should be doubled, while others suggested that the unfortunate nurse should be paid an annual sum for two or three years. The matter has been referred to the general purposes committee, and it may, we trust, be assumed that a substantial provision for a nurse who was irreparably wounded on the battle-field will receive the sanction of the authorities at Whitehall.

### THE HUDDERSFIELD NURSING ASSOCIATION.

A very powerful appeal has been made on behalf of the Huddersfield and District Victoria Sick Poor Nurses' Association. Hitherto the association has not, in the opinion of some of the leading people in the town, received the financial support which it has a right to expect from the inhabitants, but better things are hoped for in the future. The testimony of the committee to the useful work done by the nurses could not be more emphatic. Last year the nurses paid over 9,000 visits, or about 30 per day, and, as the chairman said, "thirty doors had therefore been opened every day to admit these nurses to minister to the relief of some poor suffering creatures who were unable to help themselves." It has been rightly urged that since the Sick Poor Nurses have become one of the institutions of the town "the credit of Huddersfield is at stake in not merely keeping up the work, but also in maintaining it in a state of efficiency." This is the view which should be taken by all towns where inhabitants have learnt by experience to know and to appreciate the value of the work done by trained nurses in the homes of the poor.

### A NOBLE WOMAN.

One sad event in connection with the International Congress has been the sudden and tragical death of Mrs. Ellen C. Johnson, Governor of the Massachusetts Women's Prison and Reformatory at Boston, at the house of the Bishop of Rochester in Kennington Park, where she was staying. Mrs. Johnson read a paper on "Prisons and Reformatories" at the sectional meeting on that subject on Tuesday morning, and the consequent excitement appears to have been the cause of a sudden heart attack the following morning, from which she never rallied, and died within half an hour. Mrs. Johnson's name was well known in the United States, where she was instrumental in carrying out many reforms in the treatment of women prisoners. So great was her influence over those for whom she worked that it is related that on one occasion when she offered the women prisoners at the Reformatory the choice of a "treat," and they requested a day's liberty, she had the prison gates opened and gave them their desire, only stipulating that they would return to their cells when the bell rang in the evening. She spent the day in the fields with them, and the appointed signal witnessed the unhesitating return of every prisoner to the cells, not one disappointing the trust reposed in their word of honour.

### A NURSES' HOME FOR WINCHESTER.

The governors of the Royal Hants County Hospital, Winchester, have decided to build a Nurses' Home, to reheat the whole hospital, remodel the laundry, and provide new means of escape in case of fire, at a cost of about £8,000. The Nurses' Home has been long wanted, and will, we do not doubt, greatly add to the comfort of the nursing staff. It will also enable the authorities to largely increase the private nursing staff.

### HOSPITALS WITHOUT AMBULANCES.

A striking and painful illustration of the necessity of an ambulance and stretcher being attached to every hospital, and available on application, is furnished to us by a lady who encloses her full name and address, but prefers to sign herself for publication "Sister Kathleen." She states that "the immediate removal to a hospital was imperative, and the life of the patient depended upon how this was effected. On making application to the nearest hospital, the answer was that the patient would be admitted immediately, but how he was to be conveyed there did not concern the hospital authorities. They had no ambulance or stretcher, and undertook no responsibility until the case arrived." The result was that the life of the patient was sacrificed, and Sister Kathleen thinks that many lives are thus sacrificed "for lack of a tithe of the care which patients receive when they are once in a hospital bed." It is a pity that the name of the hospital is not given us by our correspondent, but, of course, the institution cannot be in London.

### PRINCESS SALM SALM, SOLDIER AND NURSE.

The interesting announcement is made that Princess Salm Salm is about to visit England. She will certainly have a cordial reception, and many members of the nursing profession will hope that they may enjoy an opportunity of seeing her. Some of our readers will remember that before she took up the work of a Red Cross nurse, the Princess—née Rosa Leclerc—as captain, rode by the side of her husband as general of the 8th Regiment of the New York Volunteers during the Civil War, and shared his dangers and privations. Later on she went with him to Mexico and espoused the cause of the ill-fated Emperor Maximilian. Her husband lost his life at Gravelotte, and from that time for many years his widow devoted herself to the work of a Red Cross nurse. She was with the German army right through the war against France, nursing the wounded and organising hospitals with her customary fearlessness, perseverance, and enthusiasm. In 1876 she married Mr. Heneage, her present husband, having by that time attained the highest honour open to her. The order of the Verdienst-Kreuz was created for her by the Empress of Germany in lieu of that of the Iron Cross, which can only be bestowed upon a man.

### FOR INDIA'S WOMEN.

In aid of the National Association for Supplying Female Medical Aid to the Women of India a most delightful fête took place last week in the charming grounds of Kidbrook Lodge, lent for the occasion by Mr. and Mrs. Edward Edwards. At a quarter to four the Duchess of York, attended by Lady Katherine Coke and Sir Francis de Winton, arrived, and was received by the Marquis and Marchioness of Dufferin and Ava, Lord Hugh Cecil, &c. There was no formal ceremony. The Duchess of York sauntered round the stalls, temptingly spread with fruit, flowers, and sweetmeats. In screened corners lady palmists revealed mysteries in confidential undertones to numerous fair guests. Many distinguished Anglo-Indians, and those interested in Indian affairs, were present, including the Duchess of Montrose, the Countess of Annesley, Lady Mabel Annesley, Lady Helen Munro Ferguson, Lady Eva Crichton, Lady Lyall, Lady Grant Duff, &c. Tea was served in the garden, and later dinner was provided. During the afternoon a concert was given in which M. Johannes Wolff, Mr. Joseph Holtman, and Mrs. Beerbohm Tree took part. The day was brilliantly fine, the scene animated and gay, and everyone seemed rejoiced to spend money on frivolities and fruit, and not to be obliged to make purchases which they did not want. The duty of the English branch of the Association is to raise funds and select lady doctors as they may be required for the work in India. Last year a million "purdah" women were attended.

### HELPS TO TRAINED SISTERS.

At the Nursing Conference of the International Congress a foreign lady suggested that it would be well for a number of young women to train at hospitals for six months to learn a little nursing, these ladies to be available as helps to trained sisters in time of war or epidemic. "It may," says a correspondent. "interest some of your readers to know that in Germany there is a society of ladies called Johanniterinen, in connection with the Order of St. John of Jerusalem, recruited from all ranks of society, who go to the Diaconissin's and other hospitals for six months at a time (once or more frequently during their lives) to learn a certain amount of nursing and sick cookery. They were found most useful during the Franco-Prussian War, 1870-71. In Germany cooking and dispensing are in most hospitals part of a nursing sister's curriculum of education."

### SHORT ITEMS.

In view of the spread of the plague at Poona and Hong-Kong, the experiences of a sister who nursed plague patients at the Poona Hospital, which are continued this week, will be read with special interest.—On Saturday the Countess of Portsmouth reopened the Children's Ward at Tottenham Hospital, which had been closed for three years owing to lack of funds. The ward contains twelve beds and two cots, and it was immediately fully occupied.—There was a grand fête in Rosherville Gardens on Wednesday (at which the Duchess of Albany was announced to attend) on behalf of the Gravesend Hospital.—On Saturday next there will be an "At Home" at 4.30, at St. Saviour's Homes, Hendon. Delegates to the congress still in London who are interested in the subject of the care of the feeble-minded will be welcomed.—In order to make important necessary alterations and improvements the Chelsea Hospital for Women will be closed to in-patients from the middle of this month to the end of September. The out-patient department will be closed during August only.—The Liverpool School of Tropical Diseases has undertaken to give three nurses engaged by the Colonial Nursing Association a course of special training in nursing tropical diseases free of cost.—The Liverpool Ladies' Sanitary Association begins its course of training ladies as children's nurses in September. There is a good demand for refined trained women for this work. It is recommended that the domestic training should be supplemented where poss'ble by a further one in a children's convalescent home.—The sum realised by the collection and proceeds of the bazaar at the Hotel Great Central, in aid of St. Mary's Hospital, was £21,500.—On Tuesday a number of visitors went to Addlestone, the event being commemoration day of the Princess Mary Village Homes, recently described in the "Nursing Mirror." In the course of the interesting proceedings, the extension of the infirmary, built as a memorial of the Queen's Diamond Jubilee, was opened.—A Hospital Saturday was started last Saturday in Newton Abbot, the proceeds amounting to £160.

### Gynæcological Nursing.

By G. A. HAWKINS-AMBLER, F.R.C.S., Surgeon to the Samaritan Free Hospital for Women; Assistant Surgeon to the Stanley Hospital, Liverpool.

(*Continued from page* 180.)

ANTISEPTICS—(*concluded*).

THE cleansing of the hands is a matter which requires the most particular care, and is difficult, indeed, to secure; but the nurse who attends to details such as these gives an indication of her trustworthiness and is a source of immense comfort to the surgeon. She often has to wash sponges or to pass instruments at an operation, and all the care of the operator will be thrown away if the nurse has not realised the extent of her duty as to personal cleanliness. To prepare your hands it will be necessary to keep your nails trimmed very short, and not only to clean them with extreme care from apparent dirt, but to scrub them thoroughly at their edges and underneath them, and to clip away soiled skin from under the nails and tags of skin from their margin. You will then scrub the hands and arms for a good ten minutes in hot water with a new nail brush that has been soaked in 1 in 20 carbolic acid for twelve hours. The nail brush should be fairly close so that the bristles do not get under your nails and cut them. And it is well to scrub the hands under a stream of water rather than to wash and rewash them in soiled water. After this thorough and conscientious scrubbing the hands may be washed in spirits of turpentine and scrubbed again, and in order to make them still freer from germs, which are prone to linger in the deeper parts of the epithelium of the skin, it may be necessary to soak the hands and arms in a strong solution of permanganate of potash, after which they may be bleached by washing in a solution of oxalic acid and again in sterilised water, or after the scrubbing they may be soaked for two or three minutes either in 1 in 40 carbolic lotion or 1 in 1,000 corrosive sublimate lotion. After this final cleansing you will of course take care not to waste it all by handling materials, vessels, or instruments that have not been sterilised, by turning door handles, and making yourself generally useful with the odds and ends of a sickroom. The hands once cleansed must be kept in that condition for the work you have to do in connection with any operation. For less serious matters it will be sufficient to scrub the hands thoroughly with soap and water and with turpentine, and dip them in lotion.

All these may seem to be counsels of perfection, but you may be surprised to know that if, after such an elaborate cleansing and disinfection, you were to clip off a piece of skin from the hands, you would in all probability be able to grow from it a fair crop of germs. Perfection, perhaps, as regards asepsis of the hands is not to be obtained, but you can at least try, and you will run so much the less risk of conveying infection to the patient. I have known the amputation of the thigh made necessary by the carelessness of a nurse, who dressed a simple wound of the leg after attending a case of erysipelas. You may consider it an "off-chance" that damage will be done, but this "off-chance" is what makes all the difference to successful work, and it is what we are trying to eliminate from the risks of practice. Nor is this work wasted, for it saves not only the surgeon but the nurse an infinite amount of trouble in the after-management of the patient, and it is the truest economy of labour.

We shall describe the cleansing of the patient's skin when speaking of the preparations for operation.

Before being boiled, instruments are to be carefully scrubbed with a strong, new nail-brush. After dipping them in soft soap, they may be scrubbed till the lather ceases to form and then placed in the boiling water, or they may be scrubbed with dry soap or Monkey soap in the same way, and particular care must be taken with such instruments as artery forceps and clamps which have toothed ends to scrub away from the grooves of the instrument and its joints every particle of blood and dirt or rust that may adhere to them. This is not to be done in a perfunctory manner, and the nurse must understand that if she is entrusted with this duty it is a serious responsibility as well as an honour, and that on the care with which it is done will depend the success of the operation quite as much as on the skill of the surgeon performing it. Knives cannot very well be cleaned in this way, because boiling destroys the temper of the blade. If they are boiled it will be understood that the knife will have a metal handle, and the blade may be wrapped round with wool to protect it as far as possible. Perhaps the better way would be to wipe the knife carefully with a carbolised towel (1 in 20), and allow the knife to remain in a solution of carbolic acid (1 in 20) for thirty minutes before being required for use. Instruments that have been boiled as described are "sterilised"; that is, they are free from germs which may multiply and grow when introduced into the tissues. It is so simple a process that the nurse who is aware of the hour of the surgeon's visit and of the instruments he will require even for a simple examination will be wise to carry it out, and to have a quantity of sterilised water at hand for his use, as well as means of preparing readily some antiseptic solutions for which the surgeon has preference.

Where a wound is aseptic it is enough to use in connection with it instruments and water that have been sterilised. Where it is septic it will of course be necessary to use some antiseptic solution, and it will be more than ever necessary after the dressing to scrub and boil all instruments used in the process.

Dressing-trays and vessels of all kinds that are used should either be boiled or well washed in carbolic lotion after the use of soap and water, and wiped with carbolised or sterilised (boiled) towels. If the instruments and accessories are not to be used immediately they have been sterilised they must be well wrapped up or covered with dry sterilised towels. Dressings should be kept in unopened packets as far as possible; or, when the packet has been opened, those that have not been used should be carefully wrapped up in a sterilised towel and put away out of the dust until wanted. Dressings of course can be sterilised by boiling, but it is more common to use some dressing impregnated with antiseptics, which, while exerting an antiseptic influence on the parts with which it is brought into contact, is more handy for use than those which have to be sterilised on the spot, either by cooking in superheated ovens or by boiling.

These are some commonplaces of antiseptics, but we shall have to speak in more detail of these matters in describing preparations for operations of various kinds.

### Presentations.

MILL ROAD INFIRMARY, LIVERPOOL.—On the 20th of last month the staff of Mill Road Infirmary, West Derby Union, presented their late matron, Mrs. Price, with a silver tea service and a silver-mounted oak tray as a token of their esteem. Mrs. Price has been appointed to the Matronship of Brompton Hospital for Consumption and Diseases of the Chest.

THE HOSPITAL FOR WOMEN, SOHO SQUARE.—Miss Agnes Cann, who last week was the recipient of a silver tea service from the hon. medical staff on resigning the appointment of theatre and ward sister at this hospital, has been presented by the nursing staff with silver-backed brushes and comb as well as some personal gifts from the secretary and matron.

### A Year's Plague Nursing in India.

By a Sister.

### II.—AMONG THE PATIENTS.

The doctors again visited the camp in the afternoon. The temperatures of the patients about that time were usually very high, and the treatment for this was sponging and an ice-bag applied to the head with hot bottles to the feet. Cold and hot packs were often tried, but I think that this, in spite of the strictest care, was apt to induce collapse. At seven o'clock the nurses again left the camp after finishing the evening work and everything was arranged comfortably for the night. At eight o'clock the three night nurses came on duty. I must confess that night duty was gruesome, to say the least of it, and we were never sorry when our week of it was over.

Plague Patient with Bubo in the Neck.

Imagine a vast field studded with dimly-lighted sheds, each one containing plague patients in every stage of acute illness—many very ill, some dying, others actually dead, few, very few, convalescent. It was a sight sad enough to make the stoutest heart ache. Each nurse had a certain number of sheds to visit, with a book in which the names of the various acute cases were stated with the necessary treatment during the night. Never a single night passed without the sad record of several deaths. It was quite a common thing on revisiting a shed to find the patient we had left alive only a short time before lying with wide staring eyes and quite pulseless! I must confess I often felt absolute dread in drawing back the bedclothes of the patients. The camp was lighted by oil lamps, and we each carried a lantern, for the twofold reason of getting a nearer and better view of our patients and of avoiding snakes, which in that part of India are by no means uncommon. The howling of the jackals did not render night duty any the more pleasing. We returned to the nurses' shed between our rounds, and there we always partook of our nightly meals. It was sometimes very cold, and we were glad to gather round the "sigaree," which, for the benefit of my readers, I may explain was a three-legged charcoal burner standing on a large square of iron sheeting, requiring constant fanning to keep it alive. This was not so simple as it may appear, for if we used too much energy the sparks assumed such startling magnitude that there was a very grave risk of our matting shed catching fire. Our nightly cooking was done in this primitive fashion. About this time we had nearly 500 plague patients in our camp, and the constant arrival of the ambulance was a too familiar sight."

I can hardly write an account of plague nursing without giving, as far as I am able, a short description of the disease itself, and yet in doing so I know I am venturing on dangerous ground. So many people have asked me what is "plague?" is it repulsive? and what is the cause of it? Well, I think the best way to describe it is to give the symptoms of a patient suffering from it, though I may say that these differ in a variety of ways, and that although there are some unmistakable and typical signs yet the different kinds I myself have seen have been numerous. I will try and describe a typical case with the usual complications that may be expected.

### Description.

Patient is admitted either suffering from or having had a rigor. There is marked restlessness, followed afterwards by total indifference to surroundings, worried, anxious look, staggering gait, halting speech, ending sometimes in complete aphasia; hot, dry skin, temperature about 104 deg., congested conjunctivæ, furred tongue, clean in the centre, pulse quick and shallow, severe frontal headache, bubo either in the axilla, groin, or paratoid glands, and sometimes internal, delirium or unconsciousness. The complications to be expected are pneumonia (which is very common), sleeplessness, retention, complete loss of control, vomiting of a bilious nature, great exhaustion, paralysis of the glottis, marked distension, hiccoughs, suppuration infiltration, acute cerebral trouble, and heart failure.

I give an illustration of a case suffering from bubo in the axilla. The bubo is a hard swelling sometimes involving a large area. Those in the groin are the most hopeful cases, in the axilla they are more serious, as the subsequent infiltration causes most distressing symptoms. Those involving the parotid glands are nearly always fatal, indeed, universally so when both sides are affected; the latter are some of the saddest cases to nurse; the difficulty in breathing and swallowing is very great, and the fixed position of the head is most painful, as the slightest movement causes intense suffering. The local treatment for the buboes in my experience has been as follows: Ice constantly applied to the affected part, which we found very difficult to arrange, as what with the restlessness of the patient and the lack of enough ice bags, the beds were constantly wet, and it seemed impossible to keep them dry. Boracic compresses frequently renewed, linseed meal poultices, glycerine, and belladonna applied night and morning. If the bubo required surgical interference the wound was irrigated with tincture of iodine lotion, dusted over with iodoform and dressed with gauze and wool. If I may offer an opinion, I think that in those cases where the gland was

Plague Patient with Bubo in the Axilla.

issected out the result was far more favourable; this, of course, necessitated making a large deep wound, but with careful attention the wound was kept clean and healed up, whereas the long suppuration attendant on the deep incision only was exhausting to the patient, as the typical hectic temperature proved. As regards medicine, the usual remedies were stimulants and those for reducing temperature, such as diaphoretic mixture, quinine, &c., and I have also seen phenacetin given with good results. When patients were convalescent they had a tonic consisting of strychnine, quinine and iron, and so on. Hypodermic injection of strychnine and of ether were frequently prescribed. The nourishment consisted of milk, raw eggs, soup, and, when convalescent, the diet was increased to bread, rice, lentils, sago, and later on to a full diet of meat and vegetables, &c.

Now as to plague being considered a repulsive-looking disease. No, I do not think it is. Certainly it is a heart-rending sight to see the poor sufferers in a state of utter exhaustion, wild delirium, or unconsciousness, with worried, anxious look, trembling hands, and muttering speech; but there is nothing in it which the most delicate woman need shrink from approaching, and I do not think any of our nurses did shrink from it. Thirdly, what is the cause of plague? This has not yet been discovered, but there is no doubt that the lack of light and fresh air, want of sanitation and ventilation, and dirt, are predisposing causes, as might easily be seen by visiting a native quarter. The houses are built with no windows, the main entrance being a wooden door leading into a squalid room; this would again open into an inner chamber, absolutely dark, with no windows. In these two small rooms there would probably be about twelve persons asleep, with the only door closed! It is astonishing how people can live and breathe in such an atmosphere, each one wrapped, head and all, in a filthy blanket, while no fresh air or light can penetrate into the rooms where old and young, infants and aged people, are huddled together. I have often thought that the natives of India must have wonderful sight, for in passing by at night you see their houses lighted by one miserable flame produced by a thin single wick stuck into a saucer of oil.

### Queen Victoria's Jubilee Institute for Nurses.

### DISTRIBUTION OF PRIZES AND CERTIFICATES TO QUEEN'S NURSES BY H.R.H. PRINCESS LOUISE.

On Wednesday afternoon Her Royal Highness Princess Louise, Marchioness of Lorne, presented badges and certificates to Queen's Nurses at Kensington Palace. Before half-past three the neat blue uniforms had mustered in force in the fine old Council Chamber, and the Princess, looking very gracious and charming, presently arrived with the Marquis of Lorne, attended by Lady Sophia Macnamara and Colonel Collins, and accompanied by the Rev. the Master of St. Katherine's, the Duke and Duchess of Westminster, Sir Fleetwood and Lady Edwards, the Countess Cadogan, the Hon. Sydney Holland, Miss Rosalind Paget, and other members of the Council. Amongst the other guests present were:— Sir Dyce and Lady Duckworth, Major-General Sir Stanley and Lady Clarke, Mr. Thomas Bryant, the Rev. Dacre and Mrs. Craven, Miss Peter, Miss Gray, Mr. and Mrs. Archibald Williamson, Lord and Lady Blythswood, His Eminence Cardinal Vaughan, Dr. and Mrs. Adler, and Sir Squire and Lady Bancroft.

Miss Amy Graham, on behalf of the nurses, presented the Princess with a bouquet of roses, and the Rev. the Master of St. Katherine's opened the afternoon's proceedings with a brief speech, followed by

Mr. Holland, who caused much amusement by disclaiming his right to speak at all on such an occasion, saying that "there were ladies on the Council who had forgotten more about nursing than he could ever hope to know." Nurses, he had learnt, were not angels, but he found them to be honourable and devoted women, working rather harder than tram conductors to lessen the misery of the world. He asked the nurses to remember, as Queen's nurses, their responsibilities were great upon them to see to their work being carried out as the Queen herself would wish it done.

The Duke of Westminster also spoke of the high standard which it behoved Queen's nurses to keep ever before them, and moved a vote of thanks to the Princess for her presence, which was enthusiastically given by the audience.

The Marquis of Lorne replied in a few pleasant words, alluding to that great personal responsibility taken upon herself by the Queen in that very room when she met her first Council, and assuring the nurses that to the last day of Her Majesty's life her abiding interest in their welfare would remained unchanged.

Helped by Miss Rosalind Paget and Miss Leake, Princess Louise then presented silver badges (given to inspectors and superintendents of district nursing homes and county nursing associations as a mark of their office) to the following nurses:—Eleanor M. Franks, Mary E. Bullock, Annie M. Peterkin, Esther Chadwick, Alice J. Buckle, Laura A. Wing, Henrietta E. Ellis, Helen Clayton, Mary Monkhouse, Helen Sargent, Sarah A. Andrew, Emma Dudley, Else M. R. Böge, Annie I. E. Phelps, Bertha W. Hall, Florence Steel, Elizabeth Brooke, Grace Penrose, Fanny Scott, Blanche M. Glover, Helen H. Elkington, Annie Mills, Margaret Anderson Rose, Ellen B. Frederick, Charlotte Newman, Charlotte E. Youngman, Annie F. Lunn, Mary White, Martha J. Loane, Agnes M. C. A. White, Elizabeth D. Burke, Mabel Rogers, Margaret Tattam, Fanny E. Whitfield, Millicent Goodwin. Bronze badges and brassards were presented to the newly-appointed Queen's nurses, whose names will be found elsewhere, and certificates to the following superintendents and nurses, who signed no agreement to the Institute, and who have completed from two to four years' satisfactory service: Harriet Isabella Moore, Mary Katharine Taverner, Elizabeth J. Beswick, Mary Judson, Jane Beveridge Wilson, Sarah Ann Andrew, Fanny Elizabeth Whitfield, Lydia Osborne Dixon, Ethel Frances Margaret Blair, Margaret James, Mabel Hawkes, Evelyn Innes Pocock, Jane Mary Tilburn, Annie Peters, Mary Burton, Blanche Ellen Hancock, Florence Wedderburn Pritchard, Florence Beeston, Emily Cutt, Mary Goffe, and Mary Gaskell.

As the nurses received their badges and certificates they passed on into the next room, where tea was awaiting them, and the state rooms were afterwards thrown open to the visitors, who greatly enjoyed this delightful opportunity of seeing the picturesque interior of the old palace with all its interesting associations.

### Matrons' Council.

At the annual conference of this council on Saturday, presided over by Miss Isla Stewart, matron of St. Bartholomew's Hospital, it was agreed to form a provisional committee with the view of inaugurating an International Council of Nurses. Miss May Wright Sewall supported the proposal, and suggested that the organisation should be on the lines of the International Council of Women.

### Wants and Workers.

Sister Catherine, 11, Alexandra Road, Wimbledon, S.W., is anxious to hear of a spinal carriage for a boy twelve years of age. He is the son of a nurse, who is too poor to buy a new one.

Nurse Jessel, 3, Canadian Terrace, Henley-on-Thames, would be pleased to know if any reader of the "Nursing Mirror" has a bath-chair to dispose of. It is for a very stout, aged, poor woman (widow), suffering acutely from rheumatism, which, together with bad legs, render her perfectly helpless. Having been confined to her room for many weeks, she begs to be taken out. A small sum could be managed.

### International Congress of Women.

### THE NURSING SECTION.

The meetings of the International Congress were carried on with vigour to the close of the session on Tuesday, on which day Lady de Rothschild and Mrs. Leopold de Rothschild gave a garden party at Gunnersbury, and in the evening Lady Aberdeen entertained the delegates and members of committees at a farewell gathering at the Institute of Painters in Water Colours, Piccadilly.

On Friday, "Nursing" was discussed in the Council Chamber at Westminster Town Hall. In the morning, Mrs. May Wright Sewall presided, and the first paper, dealing with "The Professional Training and Status of Nurses" was read by Mrs. Grace Neill, assistant inspector of hospitals, asylums, &c., in New Zealand. Mrs. Neill reviewed the progress of nursing as a profession from the sixties onwards, and spoke of the "Standard of General Education and Age of Probationers" at the present time, advocating higher educational tests, and a later age limit, the tendency, she considered, being to allow girls to enter upon hospital life too young. The educational curriculum of hospitals, she held, should embrace a three years' course, beginning the first year with ward work and instruction in elementary anatomy and physiology; instruction in cookery, chemistry, food values, &c., to follow during the second year, while the third year's course should include the training and teaching of juniors and a foreign language. Mrs. Neill explained the working of the eight hours' system in the Wellington Hospital, and on the subject of examinations said: "In my opinion the final examination at the end of the three years' course should rest with an independent Board of Examiners, and be conducted in various localities on similar lines to the University local examination. There should be three grades of pass, and the examination should be open to any nurse who can show a matron's certificate of character having completed a three years' residence in one hospital. . . . The certificate of this proposed Central Board of Examiners should entitle to registration on their books, and when that is achieved we may leave the rest to a discriminating public." In conclusion, Mrs. Neill urged every woman, especially those having a profession, to "unceasingly to work for political enfranchisement," which when accomplished would, in her view, make a vast difference to their "interest and status."

The second paper on "State Registration of Nurses at Cape Colony" was read by Miss M. H. Watkins, and described the working of the Registration Act passed in Cape Colony in 1892. Miss Watkins considered that hitherto registration had effected a marked good by raising the standard of education for nurses, and their status; by awakening ambition; and in affording, by a published register, an opportunity to the public of knowing the qualifications of the nurses they engage. With regard to midwifery, separate examinations and certificates were given, the examinations being similar to those of the L.O.S. Miss Watkins said that the desire for registered midwives was greatly on the increase in the colony, and she trusted that the day was not far distant when the law would insist upon all midwives presenting themselves for re-examination at the end of at least every three years.

Discussion was opened by Miss Lavinia L. Dock, hon. secretary of the American Society of Superintendents of Training Schools for Nurses, who spoke of the present aspect of nursing in the United States from various points of view. Preliminary training for probationers was, she believed, about to be started by one training school after the system started in Great Britain. Registration was still in the future.

Miss Isla Stewart followed, giving an account of th extrance examination held at St. Bartholomew's, and expressing her belief that nurses should not be taught their work at the expense of the patients by being too quickly allowed to take charge of wards. Mrs. Bedford Fenwick touched on the points raised by Mrs. Neill. She commended the example set by the London Hospital in its elaboration of the preliminary training course first started at Glasgow (which, however, in her opinion, should be a self-supporting institution), and strongly advocated public examinations, training colleges for nurses, and payment for training. The latter suggestion was taken up by the Hon. Maude Stanley, who in a few practical words expressed her conviction that the nursing profession would suffer if the women of the working and professional classes were excluded from it on account of the expense consequent upon college training. Other speakers included Mrs. W. B. Rickman, Mrs. Spense, and Miss Breay.

The latter part of the morning was devoted to a paper read by Mrs. Quintard, superintendent of nurses, St. Luke's Hospital, New York, late superintendent of nursing at Camp Wikoff during the Spanish-American war, on "Naval and Military Nursing," and another by Mrs. Norrie, delegate from Denmark, upon "A Volunteer Corps of Nurses." Mrs. Quintard dwelt upon the great need for an organised system of nursing in the United States army, in peace and war, so painfully brought home to the authorities during the late fighting, and she read a draft of the Bill to secure future efficiency in this service which will presently be brought before Congress. She particularly emphasised the point that army hospitals should be governed by the same principles as civil hospitals, and that the superintendent nurse should be given due control over her staff.

Mrs. Norrie advocated hospitals opening their doors, "on certain conditions, to every young woman desirous to learn how to nurse her own sick people," for periods of three to six months, believing that in times of war or epidemic these pupils would form a useful voluntary aid to trained nurses. She thought there was no well-founded fear that such a plan would result in "educating a corps of quack nurses."

Captain Norton, M.P., opened the discussion which followed these two papers. He spoke of the English system of military nursing, condemning the insufficiency of the present training of orderlies, which he described as "half an hour's daily instruction from the superintendent sisters during one month," these men subsequently undertaking the entire night duty in the wards. He advocated an increase in the number of sisters, the appointment of lady inspectors, the maintenance of a uniform standard, and a three years' training course for the orderlies; also the establishment of a hospital ship, equipped with a proper staff. Other speakers were Mrs. Bedford Fenwick and Mrs. W. B. Rickman.

Lady Aberdeen (who opened the proceedings by reading a greeting to nurses from Miss Nightingale) presided over the afternoon session, at which Mrs. Hampton Robb's paper on "The Organisation of Trained Nurses' Alumnæ" was read by Miss Lucy Walker, superintendent of nurses, Pennsylvania Hospital, Philadelphia. Mrs. Robb enumerated the advantages of Alumnæ associations, which she considered should tend to widen feelings of fellowship amongst nurses and to raise their status. A paper by Miss Snively bearing on the same subject was read by Mrs. Bedford Fenwick, and discussion was carried on by Miss Isla Stewart, Miss Dock, Mrs. Fenwick, and Miss Lucy Walker.

The last item on the programme was a paper on "Nursing Organisations: The Victorian Order of Nurses of Canada," by Miss Scovil, of St. Paul's, Concord. Miss Scovil told of the formation by Lady Aberdeen of this order, of its organisation, the flexibility of which, while adapting it to present conditions, makes ample provision for future growth; of the work that the nurses are already doing, their difficulties, and the great need that exists for the extension of district nursing throughout the Dominion of Canada. Candidates for admission to the order must hold a diploma from a recognised training school, and receive in addition six months' training in district nursing, pledging themselves to three years' work, at a salary of about £60 per annum.

### Hospitality to Delegates at the London Hospital.

Members of the International Congress interested in nursing matters were invited to the London Hospital last Thursday afternoon. A good many responded, and found their way down to Whitechapel. The Hon. Sydney Holland (chairman) and Miss Lückes received the guests in the as yet unoccupied temporary building which is to accommodate patients while the new building operations are in progress, and Mr. Holland then explained the system upon which nursing at the hospital is conducted, and the working of Tredegar House, the preliminary training home. Tea was served in the pleasant garden, and afterwards Mr. Holland took the foreign visitors into the wards and the nurses' home, not forgetting to point out the "Garden of Eden," surely the most delicious spot in London, with its richness of grass and trees, fountains and summer-houses, presented to the nurses by the kindness of the Rev. Sydney Vatcher, vicar of St. Philip's, and chaplain to the hospital. Amongst the visitors were Mrs. Norrie and Miss Lukken, Miss Doch, Miss MacVicar, Miss Watt, Miss Bland, Miss Moir, and Miss Hughes.

### Hospitality at the Trained Nurses' Club.

The committee of the Trained Nurses' Club in Buckingham Street welcomed nurses present at the International Congress to tea on Friday afternoon, and some very interesting personalities in the hospital world were present—amongst others, Mrs. Norrie (of Denmark), Miss Lukken (matron of the large military hospital at Copenhagen), Miss Kruysse (matron of the Wilhelmina Hospital, Amsterdam), Miss Gibson, Miss Peter, Miss Hughes, Miss Swift, and Miss C. J. Wood. It was an enjoyable gathering, and afforded one of those opportunities for meeting the visitors from other lands which has been by no means the least appreciated feature of the Congress.

### Other Hospitalities.

Miss Isla Stewart, matron of St. Bartholomew's Hospital, gave an "At Home" to the nursing members of the congress "to meet the foreign delegates." On Friday evening the foreign nurses attending the congress were entertained by the Matrons' Council. Several of the delegates were taken for an excursion on the river by Mrs. A. F. Hills, president of the Vegetarian Federal Union. A reception was also given by the Young Women's Christian Association.

### Meeting of the Council of the Royal British Nurses' Association.

The first meeting of the newly-elected Council of the Royal British Nurses' Association took place on Friday afternoon. Her Royal Highness Princess Christian presided, and there were present: Mrs. Coster, Miss Thorold, Mr. Fardon, Mr. Langton, Miss Georgina Scott, Miss De Pledge, &c. After confirming the minutes of the preceding meeting, Mr. Langton, hon. treasurer, presented the financial statement for the last three months, and asked the Council to ratify the proposed disposal of the £1,552 resulting from the Café Chantant at the Hotel Cecil. By the Royal president's wish all debts of the society were to be discharged and £100 placed to the credit of the Association at the bank, and the balance invested, the proceeds, amounting, it was estimated, to some £42 a year, to be devoted to the charitable side of the Association's work. The report and suggestions were seconded by Miss Thorold, and carried unanimously. Mr. Fardon, hon. secretary, presented the report of the Executive Committee. During May and June twenty-four nurses have been registered, twenty-two elected, four have died, and eight withdrawn. The Duchess of Connaught, who has graciously consented to become one of the vice-presidents of the Association, has been asked to accept the badge of the society in gold; while Mrs. Dacre Craven, Sir Richard Douglas Powell, Bart., Sir Richard Thorne Thorne, K.C.B., and Dr. Bezly Thorne, who were elected vice-presidents at the annual meeting, were, *ex officio*, given seats on the Council. The new Council has been elected by an overwhelming majority, 542 ballot papers having been received unaltered. The following ladies, entitled under Bye-law 24 to *ex officio* seats on the Council, have expressed their willingness to serve: Miss Adam, matron, the Norfolk-Norwich Hospital; Miss Graham, matron, Mile End Infirmary; Miss Girdleston, matron, the Crumpsall Infirmary, Manchester; Miss Kelly, matron, Dr. Steeven's Hospital, Dublin; Mrs. Lawson, matron, Fir Vale Infirmary, Sheffield. The following ladies entitled under the same bye-law to *ex officio* seats on the Council have applied for membership and taken their seats: Miss Wilson, matron, Cardiff Infirmary; Miss Anstey, matron, Wandsworth Infirmary; Miss Julian, Croydon Infirmary; Miss Hopper, matron, Union Infirmary, Leeds; Miss Stewart, matron, the City of London Infirmary; Miss Wilkie, St. Luke's Hospital, Halifax; Miss Wright, matron, Town's Hospital, Glasgow. The following ladies have been appointed to represent the association as consuls: Miss Bissell, Margate; Miss Gould, matron, Hospital for Insane, Sydney, N.S.W.; Miss Foskyn, matron, Frontier Hospital, Queensland; Miss Little, sister, Royal Infirmary, Hull; Miss Mackay, matron, Frere Hospital, East London, Cape Colony; Miss Pruett, superintendent of nurses, Union Infirmary, Sunderland; Miss Ronaldson, matron, the Memorial Hospital, Buluwayo; Miss Todd, 6, The Crescent, York. Whilst the lady consuls will afford provincial and colonial nurses greater facilities for becoming members of the association, the home committee will exercise as careful a scrutiny of the evidences respecting training and testimonials as to character as hitherto, and thus so safeguard the standard of training and status of the nurse. A new card of membership has been designed, which will bear the Royal president's autograph. It is a steel engraving on fine cardboard, bearing a design in which the national emblems (the rose, the shamrock, and the thistle) and the badges of membership are introduced. An entwining ribbon is embroidered with the names of the mother country, colonies, and dependencies in which the nurses belonging to the association are working. The inscription is concise. It runs thus:—"This is to certify that............has been duly enrolled as a member of the Royal British Nurses Association." The Princess Christian signs it herself, the name not being, as on the old cards, lithographed. The cards when ready will be available for all members—new and old—of the association, and the cost has been fixed at 1s. The regulations passed at the General Council held on April 28th were confirmed; five vacancies on the list of the elected members of the General Council were filled up; and the present Executive Committee was re-elected for the ensuing year, the vacancies only being filled up by new members. By special request of the president, full details and accounts of the Café Chantant will be published in the "Nurses' Journal."

### Echoes from the Outside World.

### AN OPEN LETTER TO A HOSPITAL NURSE.

We had just begun to look upon the succession of the Duke of Connaught and his son to the throne of Saxe-Coburg and Gotha as a certainty, and many were the regrets that such a popular Prince would some day have to resign his connection with the British army and go to live in his future kingdom. But "the schemes o' mice and men" have once more gone "'a-gley.'" When the Duke recognised the fact that, having accepted the offer of the succession, he would be obliged to part from Prince Arthur, his only son, so that by residing on the spot the lad might become as much as possible a German in deed and in truth, he drew back, and asked the Diet to accept his renunciation unconditionally. But he reserved for his son the right to ascend the throne should the Duke of Albany die, or, if married, leave no male heirs. The way matters have now arranged themselves should give satisfaction to everyone. We shall keep the Duke of Connaught with us, and the Duchess of Albany will rejoice that her little son may some day become a reigning monarch. It is easy in her case for her to allow her boy to fulfil the reasonable conditions laid down by the Diet that he should at once go to Coburg to receive a thorough German education, later on to proceed to a German University and enter the German army. In this instance there is nothing to prevent the parent accompanying her child. The Duchess of Albany has since her husband's death always lived for her children, and Princess Alice's education can with advantage be finished in Germany. But how Esher will miss the Duchess with her kind thought for all sorts of people!

There is a little incident about the Duke of Albany which I happen to know to be true, and which is specially *apropos* just now. When he was much younger, before he went to Eton, he was at a large preparatory school in Surrey. There was a rule of the establishment that when ink was spilt upon the floor the boy who committed the deed should clean it up himself, and so learn to be more careful. Several times the Royal pupil had watched the amateur cleaners busy with pail and scrubbing brush obliterating the signs of their carelessness. At last on one notable day the ink was spilt again, and this time the culprit was the Duke of Albany. The misdeed was reported to the head-master, and there apparently His Royal Highness thought that, under the circumstances, the matter would end. His surprise when the fiat went forth that he, too, was to set to work and clean was comical to behold. He had evidently imagined that a grandson of Queen Victoria would be excused any such unusual task, and it took him a few seconds to realise that the wise master intended to make no difference between one pupil and another. He said nothing, but it was a very disgusted little face which could be seen as he slowly bent down and began to remove the traces of the unfortunate accident.

Womanhood in general will owe a big debt of gratitude to Mr. Gilbert Parker should his paper upon "The Housing of Educated Working Women in Large Cities," which was read towards the end of the International Congress of Women, bear practical fruit. The vivid and depressing picture which was drawn of the educated working woman in London, who, earning from 25s. to 30s. a week, possesses no home of her own, but has to fall back upon dreary lodgings or dismal, cheap boarding-houses, was all too true, and the plan propounded—should it ever be realised—would mean so much comfort to so many women. According to Mr. Parker's statement, the mansion he has in his mind will accommodate 400 professionally-employed women, who could have a double-bedded room at 8s. a week, a single one at 7s. 6d. or 5s., with free use of dining-room and drawing-room, and board at 10s. a week. Electric lighting, hot-water heating, bicycle housing, and even a professional mender of clothes on the premises are all included in the scheme, which is to be no charity in any sense, but a better-class Rowton House, to pay 5 per cent. to investors. There is every prospect that the idea will not be allowed to drop. The Countess of Aberdeen proposes, as soon as practicable, to convene a meeting of those interested in it, with a view, if possible, of taking action.

The after-season sales have begun. I had a run round the shops on Monday. Of course, they were all very full, but in the high-class shops the crowd was not nearly so formidable, because it was composed of a much nicer class of people. In one shop, where it always strikes me that the reductions are reductions in name only, the prices marked as original being so high that I wonder who would be likely to pay them, the ladies were making their purchases clad in the most gorgeous attire, many wearing light silk dresses trimmed with real lace, accompanied by the lightest kid gloves. They looked as if they had made a mistake, and had fancied it was going to be a garden party rather than a sale at an ordinary London shop, though I know it is highly correct now to wear such "smart frocks" in the morning. I am afraid I still hold the old-fashioned belief that the canons of good taste decree the gown should suit the occasion; but I should be looked upon as a hopelessly "frumpish" person to object to silk and satin for morning wear. One of the shops had been most considerate for the comfort of their visitors. In every department there were huge blocks of ice, with cool looking ferns at the base. The result was that the rooms, though crowded, were always delightfully cool. Just one hint, if you have a pound or so to lay out at sale time. If you can keep a cool head, and only buy what you want, money spent at sales is money well invested. Only don't go to any but the best shops. The sales, as a rule, are more genuine and more inclusive than in the smaller ones, so that if you can wait to make your purchases till July or January you can buy a much better material for the same price you would have paid for an inferior article at another time.

The time of holidays is rapidly approaching, and some of us who are looking forward with joy to trips in Ireland or on the Continent are also remembering with apprehension and dread that there is the awful crossing to be got through first, with its attendant horror of *mal-de-mer*. Most of us have long ago given up any belief in remedies, and after having submitted to starving a couple of days beforehand, lying on our backs all the time of the voyage sucking small pieces of ice, sipping champagne every five minutes, or taking noxious drugs every half an hour, we have resigned ourselves to the inevitable, and looked on sea-sickness as one of the ills to which flesh is heir. But now a German doctor has discovered what he believes to be an infallible remedy, although I cannot quite see how it will benefit a traveller by a night boat. All you have to do, according to this wise man, is to procure a pair of red spectacles. (I conclude you will be obliged to have them made to order, as I have never seen such things at an optician's.) The red colour is said to have such an exciting influence on the brain that no sea, however rolling, if viewed through this medium, will have any effect on a head with the circulation thus quickened. At any rate, the experiment is worth trying, as it can do little harm, and it may possibly throw a most desirable rosy hue over all the marine surroundings.

### Miss Burns' Wedding.

By a Correspondent.

It was a very pretty scene at St. Margaret's, Westminster, on Saturday, when Miss Burns and Mr. Lewis Harcourt were married. The church was quite full of the friends of the bride and bridegroom, and was simply but charmingly decorated with white flowers and palms. A large number of both Houses of Parliament were present. The bride, who was given away by her brother, Mr. Walter Burns, was attended by ten bridesmaids prettily dressed in white with touches of mauve, and carrying bouquets of blush roses. The bride's dress of white satin and Brussels lace was elegant and simple, very suited to her graceful and dignified style. I do not intend to enter into a long description of the many beautiful dresses worn, but I cannot omit to mention the very becoming costume, wholly of white, worn by the bride's mother, Mrs. Burns. The deviation from the heavy colours usually worn on such occasions was most successful, and it seemed most fitting that when for a moment the widowed mother put aside all trace of mourning that she should have chosen to appear in white, instead of adopting a compromise. The absent father was in the thoughts of not a few of the assembled company, with the regret that he was not spared to witness the happiness of his daughter. The marriage ceremony was performed by the Bishop of Winchester, who delivered a short address, and the service, which was choral, was most impressive. Handel's beautiful anthem, "He Watching over Israel," filled the church with soft melody whilst the bridal party resorted to the vestry, and during the service the hymns "O Perfect Love" and "Praise the Lord, Ye Heavens Adore Him" were sung. The Wedding March from "Tannhaüser" was played as the bride and bridegroom left the church. The greeting they received from their friends was most hearty, and it was delightful to hear the kindly words expressed on all sides amongst the assemblage. This was no gathering of an indifferent Society crowd. The genuine respect and esteem felt for the parents of both bride and bridegroom, added to the friendship which they have each gained for themselves, united all those present in the warmest wishes for a happy future, which should surely be theirs. Even on an occasion when a little self-absorption would be most excusable, evidence of the sweet kindliness of the bride were not wanting. A little group of nurses, their hands full of pretty floral wedding favours, were talking together on their way out of the church. "Yes," said one, "in spite of her happiness, and in spite of all her friends surrounding her, she turned to me as she came out of the vestry and gave me a kiss. She is a dear," with which the other nurses who had been invited cordially agreed. None of those who had ministered in sickness were forgotten in the time of rejoicing, a fact which, little as it needs it, will help to cement the bond of sympathy between the family to which Miss Burns belongs to the large army of nurses for whom they have done so much. The large company who assembled at 69, Brook Street, after the marriage ceremony had an opportunity of seeing the testimony of good-will presented to Miss Burns by the nurses of the Pension Fund, and which found a place amongst the many beautiful gifts displayed in the ball-room. The subject of wedding presents is always dear to the feminine heart, so I must mention a few of the beautiful things which formed part of the 700 gifts received by the bride and bridegroom. Perhaps the two most interesting were—the one, a glorious necklace of diamonds of single pendant stones, left as a wedding gift to the bride by her grandfather, which had formed part of the Crown jewels of France; and the other, a beautiful portrait of Romney by himself, a present from the bride's mother. She also gave her daughter a magnificent crown of diamonds, valuable lace, and other gifts, and Mr. Walter Burns gave his sister an exquisite coronet and suite of turquoises and diamonds. Amongst the presents to the bridegroom was some interesting old family plate and a dessert service of Royal blue Derby china from the Liberals of Derby. Space forbids me dwelling any longer upon the many magnificent and beautiful offerings. I noticed a very much larger proportion of books than usually find a place amongst wedding gifts, and they formed an interesting and beautifully-bound little library. The ball-room, and indeed the whole of Mrs. Burns' house was charmingly decorated with flowers, which were placed in every available space and corner. The window-sills were massed with crimson and pink roses, and every fireplace was filled with tall white lilies. Mrs. Burns received the large company of distinguished guests at the top of the fine staircase, and in the first reception-room friends had the pleasure of greeting the bride and bridegroom, who left late in the afternoon for Nuneham Park, a beautiful residence on the river near Oxford, lent them by Mr. Aubrey Harcourt, cousin to Mr. Lewis Harcourt.

### Queen Victoria's Jubilee Institute for Nurses.

Her Majesty has been graciously pleased to approve the appointment of the following as Queen's Nurses, to date July 1st, 1899:—

England.—Marion Ashwell, Mary Amelia Pilliet, Edith Margaret Eleanor Lownds, Edith Harvey, Mary Isabella Everall, Julia Elizabeth Pegg, Rosalina Mary Jane D'Arcy, and Katharine Isabel Perkins, London; Fanny Maria Martin, East London; Alice King and Caroline Trotman, Chatham; Mabel Harrison, Strood; Evelyn Mary Smith, Hayes; Susan Jessie Grant, King's Walden; Emily Reid, Braughing; Sarah Monica Johnson, Carshalton; Jennie Edden and Mary Winifred Chexfield, Windsor; Annie Gertrude Davidson, Kingston-on-Thames; Lucy Elizabeth Pierce, Leamington; Lizzie Odell Carter and Frances Harriett Warter, Reading; Margaret Rebecca Scarth and Johanna Sophia Schilt, Grimsby; Annie Keeler Roalfe, Cheltenham; Frances Annie Howcroft, Southampton; Gertrude Hesser Burrell and Alice Helen Mary Childe, Brighton; Mary Anne Benbow Cave, St. Mary Extra; Annie Gurd, Freshford; Edith Sarah Topham, Ditchingham; Miriam Phillips, Wisbech; Mary Annie Manlove, Colchester; Ellen Alice Hammersley, Leeds; Lily King and Annie Williams, Birmingham; Clare Harris, Melbourne; Kathleen Scott O'Connor, Stafford; Mary Rose Kendrick, Bedford; Eliza Ellen Birnie, Hull; Florence Blackhurst, Bishop Auckland; Lizzie Lineham, Zoe Mai Brooke-Jones, and Florence Elizabeth Nash, Blackburn; Mary Adela Flora Patterson, Rochdale; Coral Archer, Droylsden; Florence Marion Leshaw and Lottie Harvey, Warrington; Kathleen Synge Townshend, Minnie Canning, Gertrude Lucas, Emma Sykes, Mary E. S. A. Hirst, Frances Bradbury, and Emily Maria Farrar, Liverpool; Sarah Robinson, Manchester; Annie Elise Gertoff Pringle, Millom; Mary Agnes Jefferson, Barrow-in-Furness; Frances Rachel Walter, Ellol; Eva Markby, Exmouth; Amy Graham, Haydock; Maude Oldacre, Rusholme; Edith Augusta Bellamy, Shrewsbury; Emily Macmullen, Cheltenham; Lucy Hill.

Wales.—Mary Evans, Cardiff; Jeanie Williams, Rhayader; Shennie Roger Lewis, Harlech; Emma Wilhelmina Irby, Conway; Annie Elizabeth Gill, Amlwch; Lillian Genetta Newcomb, Nantlle Vale.

Scotland.—Christina Russell Ker, Aberdeen; Georgina Williams, Beith; Susan Alexander Mackinnon and Jane Constable Millar, Glasgow; Isabella Paul Dow, Paisley; Elizabeth Wark, Blantyre; Mary Selkirk, Wishaw; Margaret Altmont, Alexandria; Eva Lane Brown, Bothwell; Agnes Greig, Kilburnie; Jane Souter, Galston; Sara Gradwell, Larbert; Barbara Scott, Thornhill; Hamilton Murray, Dumfries; Margaret Day, Blairgowrie; Janet Wylie Hadden, Tobermory.

Ireland.—Margery Montgomery, Marion Hogg, Emma Graham, and Frances McCartie, Dublin; Margaret A. Carew, Banbridge; Mary Anne Massey, Cushendall; Cornelia Frances Dowling, Galway; Annie Henry, Swords; Elizabeth Annie Cole, Ballinamallard; Anna Parr, Kilkenny.

### Everybody's Opinion.

[Correspondence on all subjects is invited, but we cannot in any way be responsible for the opinions expressed by our correspondents. No communication can be entertained if the name and address of the correspondent is not given, as a guarantee of good faith but not necessarily for publication, or unless one side of the paper only is written on.]

### CHEYNE-STOKES RESPIRATION.

"A NURSE" writes: A correspondent's account of Cheyne-Stokes respiration in a case of carcinoma which I read in THE HOSPITAL "Mirror" reminds me of a very interesting case I had some two years ago or more. The patient was an old gentleman of wonderfully strong constitution suffering from apoplexy. For over three months he had Cheyne-Stokes respiration day and night, the intervals of breathing and of silence varying only a few seconds from time to time. Often the breathing would increase in intensity until it could easily be heard by people on the floors above and below his bedroom, and the silence which intervened at such times seemed deathly. The patient had been ill for a long time before I went to him, and after being nursed for fourteen weeks died a hard and painful death. I have been nursing many years in hospital and private houses, but never before or since have I seen a case of Cheyne-Stokes respiration last so long. It would be very interesting to have the experience of other nurses on this subject.

### "DON'T."

"ANOTHER OLD PRO" writes: I think with the author of "Don't" that "A Very Old Pro" is not blessed with any sense of humour, as she is such a "very old pro" that she has forgotten her "pro" days. There are not many "pro's" who would not see the hidden humour in most, if not all, the "don'ts." I laughed heartily over some of them, having keen recollections of being "sat upon" (most "pro's" will understand that term) for doing some of the "don'ts." Perhaps "A Very Old Pro" had some kind friend who gave her a few hints as to what a new "pro" may not do, as she does not grasp the meaning of many of the "don'ts"; it seems they could not have been impressed on her in the way they are on most "pro's." I'm sure the majority of "pro's" who enter hospital life perfectly strange to all its unwritten rules soon find out by a few well-directed snubs what they may "not" do. What a good thing it is to be blessed with a keen sense of humour. I'm sorry for "Very Old Pro" that she apparently has so little. I'm thankful to say I possess a large vein of it, for it does help one so much in the ups and downs of a nurse's life to be able to see the amusing side of things, as well as the dark side.

### "TALKING SHOP."

"L. G. M." writes: May I most heartily echo Alice Fitzgerald's sentiments? It is perfectly shocking to hear the way in which some nurses—and not, alas! only young nurses—discuss their cases and hospital work generally. They have no regard whatever for the "fitness of things," but talk of all sorts of cases, every kind of "shop," on the tops of omnibuses, in restaurants, in the street, to the great disgust of all hearers. A friend of mine once heard two nurses discussing a case in detail on the top of an omnibus in such a way as to make the driver of the omnibus comment disgustingly and sarcastically upon them. They bring discredit upon their whole profession, and they must infallibly lose their own sense of modesty and self-respect. No wonder that you hear people say, "Oh! a nurse loses all sense of modesty." But she need not do so. She ought not to do so if she will only regard the simple rules of decency and order and bridle her tongue. Perpetual "shop" of any sort is wearisome to the listener—hospital "shop" should never be talked in the presence of the laity. Indeed, there is often a great deal too much of it even between the walls of hospitals.

### THE READING SOCIETY.

"A CONSTANT READER" writes: May I be permitted to offer a suggestion regarding a reading society for nurses. As a private nurse I find I get more out of touch with surgical matters even than medical, and I have frequently heard others engaged in private work say the same. Could not "L. G. M." kindly include a paper on modern surgery amongst her proposed rules? In that case she will, I am sure, supply a want long felt by private nurses.

"MISS L. G. MOBERLY" writes: So many nurses have already sent in their names for the proposed Reading Society that I am starting it at once. Efforts will be made to arrange about a lending library as soon as the number of nurses who join is known. Also a lady, much interested in the matter, has offered to help towards money prizes, and if other persons who take an interest in nurses would likewise assist, quarterly prizes, I believe, could easily be arranged. The following are the rules: 1. One book only a quarter will be kept, by desire of many members. 2. One paper of questions upon the contents of the book will be set every quarter. 3. One paper upon general nursing subjects will also be set each quarter. 4. Subscriptions of 2s. 6d. per annum to be sent to Miss L. G. Moberly, 24, Portland Place, W. 5. No letter can be answered unless a stamped envelope be enclosed.

### Appointments.

THE COTTAGE HOSPITAL, BROMLEY, KENT.—Miss Annie Carter has been appointed Matron. She was trained for three years at the Devon and Exeter Hospital. She has since held sisters' posts at the Suffolk General and at Lewisham Infirmary. Since June, 1898, Miss Carter has been matron of the Joint Isolation Hospital, Mogden, Isleworth.

POPLAR AND STEPNEY SICK ASYLUM, BROMLEY, MIDDLESEX.—Miss Susan Milne has been appointed Superintending Night Nurse. She was trained at the North Riding Infirmary, Middlesboro', and has since been staff nurse at St. Saviour's Infirmary, East Dulwich, and sister at the above infirmary four years.

CHESTER-LE-STREET UNION WORKHOUSE INFIRMARY.—On June 22nd Nurse Grace Branson was appointed Superintending Nurse. She was trained at Stockton and Thornaby Hospital, and has since been nurse at Chester-le-Street Union Infirmary and at Stockton and Thornaby Hospital.

GREAT YARMOUTH HOSPITAL.—On June 26th, Miss Ethel M. Harrison was appointed Sister. She was trained at the General Infirmary, Northampton, and was afterwards charge nurse at the same place.

ADELAIDE HOSPITAL, SOUTH AUSTRALIA.—On May 12th Miss Winifred Beatrice Norman was appointed Charge Nurse. She was trained at the Geelong Infirmary, Victoria.

### Minor Appointments.

METROPOLITAN CONVALESCENT INSTITUTION, BROADSTAIRS, CHILDREN'S BRANCH.—July 4th—Miss Mary Glenton-Kerr was appointed Assistant Matron. She was trained at the Evelina Hospital for Sick Children, and Leeds General Infirmary. Her previous appointments have been ward sister and night superintendent at St. Mark's Hospital, City Road; charge nurse—children's ward, and theatre nurse at Leeds Infirmary.

NORTHERN CONVALESCENT FEVER HOSPITAL, WINCHMORE HILL.—On June 23rd Miss Helena Butler was appointed Superintendent Night Nurse. She was trained at St. Bartholomew's Hospital (1891-1896), and from that time to the present has been charge nurse and night superintendent at the hospital, Johannesburg, South Africa.

GRAVESEND WORKHOUSE INFIRMARY.—On June 15th Miss Martha Ridgway was appointed Superintending Nurse. She was trained at the Greenwich Union Infirmary.

### Death in Our Ranks.

WE regret to announce the death of Miss Florentina Finlay, third daughter of the late Sir Thomas Finlay, Kt., J.P., who passed away suddenly on the 22nd inst. at 13, Lithos Road, South Hampstead, after twelve years' continuous work. She served as night superintendent at the Belfast Royal Hospital, and was obliged in December last to resign the post of lady superintendent at the Newark-on-Trent Hospital on account of failing health.

### For Reading to the Sick.

"OUR STEWARDSHIP."

**Verses.**

GOD bends from out the deep, and says,
"I gave thee the great gift of Life;
Wast thou not called in many ways?
Are not My earth and heaven at strife?
I gave thee of My seed to sow—
Bringest thou Me My hundred-fold?"
Can I look up with face aglow,
And answer, "Father, here is gold?"—*Lowell.*

No stream from its source
Flows seaward, how lonely soever its course,
But what some land is gladden'd! No star ever rose
And set without influence somewhere; Who knows
What earth needs from earth's lowest creature? No life
Can be pure in its purpose and strong in its strife,
And all life not be purer and stronger thereby.

The spirit of just men made perfect on high—
The army of martyrs who stand by the throne,
And gaze into the Face that makes glorious their own;
Know this, surely at last! honest love, honest sorrow;
Honest work for the day, honest hope for the morrow;
Are these worth nothing more than the hand they make weary,
The heart they have saddened, the life they leave dreary?
Hush! the sevenfold Heavens to the voice of the Spirit
Echo: "He that overcometh shall all things inherit."
—*Lytton.*

**Reading.**

Each single life is seen in the Incarnation to be, in the Divine plan, an element in the Body of Christ.—*Westcott.*

The character of a generation is moulded by personal character.—*Westcott.*

Look at the man to whom his Lord had given one talent! He could not bear the thought of using his talent according to the will of Him from whom he had it, and therefore he chose to make himself happier in a way of his own. "Lord," said he, "I knew thee that thou wast a hard man, reaping where thou hast not sown, and gathering where thou hast not strawed." But his Lord having convicted him out of his own mouth, despatched him with this sentence, "Cast the unprofitable servant into outer darkness." Here you see what this man secured by not acting wholly according to his Lord's will. It was, according to his own account, a life of murmuring and discontent. "I knew thee," said he, "that thou wast a hard man." It was a life of fears and apprehensions. "I was," said he, "afraid it was a life of vain labours and fruitless travails." "I went," said he, "and hid my talent," and after having been awhile the sport of foolish passions, tormenting fears, and fruitless labours, he is rewarded with darkness, eternal weeping, and gnashing of teeth. Look at he man with his five talents! Here you see a man wholly ntent on improving his talents; his work prospers in his hand; the blessing of five becomes the blessing of ten talents, and he is received with a "Well done, good and faithful servant; enter thou into the joy of thy Lord."—*Bogatsky.*

### TRAVEL NOTES AND QUERIES.

NORMANDY OR BRITTANY (Subscriber).—I wish you had told me how much you could spend. You could spend a fortnight at St. Servan, in Brittany, and two nights at Mont St. Michel, and pay your journey, for £7 each. Full particulars of this trip, in two articles, appeared in the "Mirror" a few weeks ago—"A Cheap Holiday for Nurses." There is no expense attendant upon taking a cycle into France, except 50 centimes for a *permis de circulation* at the Custom House, so that perhaps it would be as well to hire one in England and take it out. I believe you can hire at Dinard, across the Bay, but not cheaply. An alternative trip would be to the Ardennes (good cycling ground), making headquarters at Dinant, on the Meuse. Expense almost identical, perhaps a trifle more. A third, on the Rhine, making your headquarters at Konigswinter or Bacharach. This, too, would not be expensive. If you will write to me again and say which you settle on I will give you more particulars.

### Notes and Queries.

The contents of the Editor's Letter-box have now reached such unwieldy proportions that it has become necessary to establish a hard and fast rule regarding Answers to Correspondents. In future, all questions requiring replies will continue to be answered in this column without any fee. If an answer is required by letter, a fee of half-a-crown must be enclosed with the note containing the enquiry. We are always pleased to help our numerous correspondents to the fullest extent, and we can trust them to sympathise in the overwhelming amount of writing which makes the new rules a necessity.

Every communication must be accompanied by the writer's name and address, otherwise it will receive no attention.

*Hearthstone.*

(126) I shall be grateful if any of your readers can tell me of a stone or powder which will replace the ordinary hearthstone, and wear off less easily. We have huge corridors, and several of our ward floors being polished, feet-marks from the hearthstone are disastrous to keeping them in good order. The corridors and steps must be cleaned with something of that sort, or the stones look so wretched.—*Matron.*

We cannot give the name of any other powder for use instead of hearthstone, but a matron has kindly given us directions how to use the hearthstone with the best effect. The old stone is washed off clean every morning, and the fresh stone rubbed in thoroughly and smoothly with a flannel. After this the flannel is rinsed and wrung dry, and a final working in of the stone given. All irregularities being removed, a finish is attained by even strokes backwards and forwards. The real secret is working the hearthstone smoothly into the stone, instead of laying it on superficially and allowing it to dry.

*Children's Hospitals.*

(127) Would you kindly inform me whether there is a special paper in which there are advertisements for probationers for children's hospitals? I have been taking THE HOSPITAL for some time, but have not as yet seen an advertisement. I have the book entitled "Nursing Profession: How and Where to Train."—*Anxious.*

The "Mirror" is acknowledged to be the best medium for nursing advertisements, but there is generally no need for children's hospitals to advertise their vacancies, as the number of applicants is so great. Your best chance of success, therefore, is to select the hospitals which suit you, and ask the matrons to put you on their list of candidates.

*Consulting a Specialist.*

(128) Could you inform me what is the best course to take in regard to a country patient going to a London hospital to have an examination of the nose? Is it necessary to get a letter of recommendation, or would a letter from your own doctor be sufficient? What hospital would you advise for the nose and throat?—*Pembrokeshire.*

Most of the large London hospitals have departments in special branches of medicine and surgery. The Central London Throat, Nose, and Ear Hospital, Gray's Inn Road, W.C., and the London Throat Hospital for Diseases of the Throat, Nose, and Ear, 204, Great Portland Street, W., are the two devoted to your case. Both are free. Your best plan is to ask the medical attendant to write and make arrangements with the specialist he would like the patient to be under before he leaves home. Any medical man can recommend a patient to the consultations held at the London Polyclinic.

*Massage.*

(129) I am a probationer in a cottage hospital, receiving a small salary, because, being a midwife, I agreed to help the district midwife when necessary. I am also a masseuse. A patient has been sent daily to the hospital for treatment by me. I have also been requested to teach one of the other nurses massage to enable her to give the treatment in the district. I had to pay for my certificate. Are the authorities justified in expecting one to do this work and teach other nurses gratis, the hospital receiving the benefit?—*Probationer.*

You would certainly only be required to do the work you undertook. Any further work should be a matter of mutual arrangement.

*Disinfection.*

(130) Will you advise me as to the best preparation of carbolic for disinfecting clothes in cases of scarlet fever? Is it necessary to have it 1 in 20? I have found considerable difficulty in private houses in obtaining a sufficient quantity. Is there any cheaper method of preparing it than in buying the prepared lotion?—*Economy.*

In regard to the disinfection of clothes in cases of fever—scarlet fever, typhoid fever, measles, &c.—it has to be remembered that it is not the carbolic or other chemical that is the real disinfectant. What these things do is to prevent the development of the germs during the interval which must elapse before the clothes are subjected to real disinfection by the heat to which they are exposed when they are boiled. It is not, therefore, absolutely necessary to use a full strength disinfectant for the purpose. One in 40 carbolic is sufficient. In regard to typhoid fever, which may be taken as a type, "Allbutt's System of Medicine" says: "The bed linen, blanket, and body linen of the patient should be changed at once when soiled. They should be placed in a sheet soaked in carbolic acid (1 in 40), and afterwards kept for some hours in carbolic acid solution of the same strength. Before they are sent to the laundry they should be well boiled." It is not generally worth while to buy the carbolic solution ready prepared. For purposes of disinfection the common (No. 5 Calvert's carbolic acid) acid is good enough. One ounce by measure of this should be added to a quart of hot water, and well stirred up by aid of a spoon—not with the hand—to ensure solution. For the disinfection of stools a stronger solution—not less than 1 in 20—is necessary, and in the preparation of this stronger solution boiling water is necessary, and still greater care is required to ensure that the acid is completely dissolved. The poisonous nature of carbolic acid must be always borne in mind, and the bottle must be plainly labelled, and kept under lock and key. For the disinfection of the nurse's hands corrosive sublimate solution (1 in 1,000) is the best, as it does not roughen the skin as carbolic acid does.

## Figures and Tables

**Figure f1:**
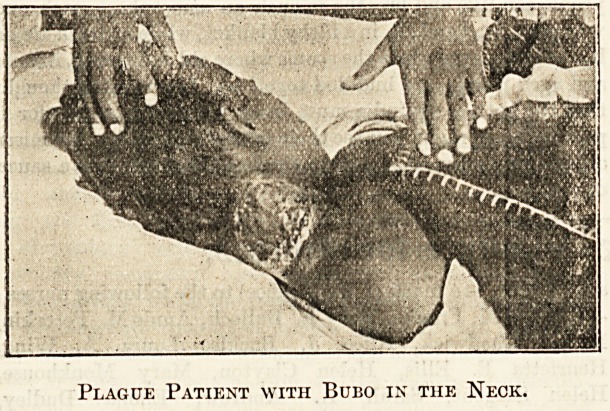


**Figure f2:**